# The Use of Family History in Primary Health Care: A Qualitative Study

**DOI:** 10.1155/2013/695763

**Published:** 2013-07-14

**Authors:** Sarah Daelemans, Jan Vandevoorde, Johan Vansintejan, Liesbeth Borgermans, Dirk Devroey

**Affiliations:** Department of Family Medicine, Vrije Universiteit Brussel (VUB), Laarbeeklaan 103, 1090 Brussels, Belgium

## Abstract

The aim of this study is to describe how Belgian family physicians register and use the family history data of their patients in daily practice. Qualitative in-depth semistructured one-to-one interviews were conducted including 16 family physicians in Belgium. These interviews were recorded, transcribed, and analysed. Recurring themes were identified and compared with findings from the existing literature. All interviewed family physicians considered the family history as an important part of the medical records. Half of the surveyed physicians confirmed knowing the family history of at least 50% of their patients. The data on family history were mainly collected during the first consultations with the patient. The majority of physicians did not use a standardised questionnaire or form to collect and to record the family history. To estimate the impact of a family history, physicians seldom use official guidance or resources. Physicians perceived a lack of time and unreliable information provided by their patients as obstacles to collect and interpret the family history. Solutions that foster the use of family history data were identified at the level of the physician and also included the development of specific instruments integrated within the electronic medical record.

## 1. Introduction

### 1.1. Medical Genetics and Family History

Medical genetics is a rapidly evolving area of medicine. Including the completion of the Human Genome Project in 2003, our knowledge about the hereditary aspect of various diseases has vastly increased. This genetic background is important not only for single-gene disorders such as cystic fibrosis, but also for multifactorial diseases such as hypertension, diabetes mellitus, cardiovascular problems, mental disorders, and various malignancies [1, 2]. For many frequently occurring multifactorial disorders, a positive family history is a known risk factor [3, 4].

A family history for a frequently occurring disorder such as diabetes, cardiovascular disease, and various cancers is associated with a relative risk that is two to five times higher than that of the general population [4–6]. A positive family history reflects an inherited genetic susceptibility and also shared common environmental factors and behaviour within the family [4, 5, 7–9].

Family history is associated with a higher risk of disease when multiple family members are affected, when the affected family members are first or second degree relatives, and when the disease occurs at a young age [4, 5]. Basic or more specific risk provisions can be used to develop specific guidelines that are customized for each risk group [10]. In high-risk patients, genetic testing and genetic counselling can be considered. Here, the family history is useful to evaluate the penetration of a possible mutation in the family [8, 11]. Subjects with an increased risk should also benefit from a customised screening and prevention programme, similar to those for breast cancer and colorectal cancer. Risk assessment and classification are unique for each disease. The family history should be reassessed regularly, as family history can change over time [7].

### 1.2. The Role of the Family Physician

Many studies suggest that together with the progress in medical genetics, the importance of primary care with respect to the prevention and early detection of heritable disorders will grow significantly [3, 12–15]. In future, family physicians will increasingly play a role in genetic counselling using skills such as (1) collecting detailed data on family history, (2) identifying, informing and following persons at risk, (3) coordination of care for individual patients and their family, and (4) providing appropriate psychological support [3, 15]. The family physicians occupy a privileged place in health care; they have unique relationships with both patients and other family members, providing them with some foreknowledge about the family medical history.

Several studies have shown that the vast majority of family physicians consider the family history as important, but there remains much room for improvement when it comes to regular survey, proper registration, and interpretation of this history [3, 16–19]. Underestimation of the risk can result in missed screening and diagnostic opportunities, while overestimation of risk can lead to overuse of medical services and unnecessary prophylactic treatments [20].

### 1.3. The Gold Standard for the Collection of Family History

Several studies provide a description of the ideal collection of family history data [1, 4, 7, 16, 19, 21]. The questioning of the patient is usually done in a personal interview, eventually preceded by a written or telephone survey in which the patient is encouraged to contact relatives in order to gather or confirm family information of at least three generations of relatives, including grandparents, uncles, and aunts, preferably displayed in a genealogical tree.

### 1.4. Barriers

For a family physician, the collection of the family history is a time-consuming activity which turns out to be difficult during regular encounters with patients [19]. Many family physicians experience a lack of time to be a major barrier to obtain an accurate family history [1, 16, 19, 22]. Other barriers perceived are a lack of accurate information as provided by the patient on medical conditions that occur in his/her family and the fact that many family physicians doubt their knowledge and skills to correctly inquire and interpret the family history and to guide the patient further on [1, 16, 17, 22, 23]. Family physicians need clear guidelines and recommendations for the collection and interpretation of the family history, risk assessment, and any subsequent referrals to secondary care [16, 19, 23].

Currently, there is no consensus on how family history should be inquired about in primary health care. The three generations comprising family tree, drawn up by a geneticist, can be seen as the gold standard, but it is not proven whether this approach is also cost-effective in primary care [1, 8].

In Belgium, the scientific organisation of family physicians “Domus Medica” recently developed the “Health Guide.” It is a preventive instrument that aims at an annual completion of a health questionnaire. The family history concerning heart disease, diabetes, breast and ovarian cancer, and colon cancer or polyps is inquired. The family physician uses the completed questionnaire to establish an individual prevention plan for the patient [24]. At the onset of our study, this Health Guide was made available to family physicians in Belgium.

### 1.5. Aims of the Study

This study aims (1) to examine the attitude of Belgian family physicians to the use of family history in primary health care, (2) to describe the way these physicians are currently inquiring and recording family history, and (3) to describe the weaknesses and opportunities of the registration of the family history.

## 2. Methods

### 2.1. Recruitment

A database with all family medicine training supervisors of the Vrije Universiteit Brussel (*N* = 200) was stratified by gender, years of experience as a physician, working place (urban and rural), and practice type (group, duo, or solo practices). These family medicine training supervisors are common family physicians who reflect family physicians in general. They are no academics but supervise the training of students in their own practice. They do not receive a financial compensation such as a honorarium or payment of expenses. 

Recruitment was phased. Firstly, all physicians received information about the study by e-mail and were invited to participate. Ten physicians participated in the study, and data analyses were made after every interview. Two authors (S. Daelemans and D. Devroey) monitored data saturation. When no new relevant knowledge was being obtained from new participants, data saturation was considered. Data saturation was not obtained after the first ten interviews. Subsequently, nonresponders were contacted by telephone, seeking a proportional participation of physicians according to the stratified database of supervisors. Recruitment was closed once saturation of data was obtained.

### 2.2. Interviews

The qualitative in-depth interviews were conducted in the period between November 2011 and February 2012. The semistructured questionnaire for the interviews was initially based on the questions used in the study conducted by Mathers et al. [13]. In this study, the emphasis was made on family history, genetics, and genetic counselling in primary health care.

After a pilot interview with one of the family physicians of the Department of Family Medicine of the Vrije Universiteit Brussel, the questionnaire was adapted and the emphasis was placed on the use of a family history. The questionnaire was further refined to a final list of 23 fixed questions on *how and when the positive and negative family history was inquired and recorded, the closeness of the inquired relatives, risk management, reliability of the family history, importance, and possible obstacles*. The pilot interview was not included in the final data because major changes were made to the initial questionnaire.

Interviews were conducted according to the preference of the participating physician during a personal interview or by telephone. During the interviews, a structured questionnaire was used. It was regularly extended with additional questions derived from the information collected during earlier interviews. The duration of interviews ranged between 9 and 33 minutes.

### 2.3. Approval of Ethical Comity

Before the start of the study the protocol was approved by the Commission for Medical Ethics of the University Hospital of the Vrije Universiteit Brussel. 

Before the start of each interview, the participant was informed about the methods and the aims of the study and he/she could ask any questions. Participants who were personally interviewed signed an informed consent sheet. Participants who were interviewed by telephone gave verbal consent, which was recorded. All the latter participants were informed and aware that the interviews were digitally recorded.

### 2.4. Data Analysis

Thematic analysis was done after each interview to assure the introduction of supplementary questions in subsequent interviews and to judge the saturation level. The digital recordings were transcribed literally. The members of the research team read all transcripts immediately after each interview and the interviewer (S. Daelemans) subsequently performed the data analysis. During data analysis, references were made to the recordings and transcripts. Thereafter within-case coding and categorisation was done. Finally, a cross-case thematic framework and a thematic grid was made allowing us to make cross-case comparisons based on sample characteristics and allowing us to check the within-case contextual validity [25]. A similar analytic method to that of Mathers et al. was used [13].

## 3. Results

### 3.1. Demographic Information

Sixteen physicians from 16 different primary care practices participated in the study. A small majority (9/16) of participants were females. The number of years of experience as a family physician ranged from 4 to 40 years. Seven of the participants worked for at least 20 years as a family physician. The average experience was 19.4 years (standard deviation = 11.7). Three participants followed some kind of training in relation to family history or genetic disorders. There was an equal distribution of physicians working in urban and rural practices. Four of the surveyed physician worked alone, three worked in a duo practice, and the other nine physicians worked in a group practice. All characteristics of the surveyed physicians, their practice and patient population can be found in Table 1.

### 3.2. For What Proportion of Patients Was Family History Ever Checked?

Most participants claimed that they inquired about the family history for at least 50% of their patients. They inquired about the family history for all or almost all patients.

Many of the physicians that inquired about the family history of at least 50% of their patients had 20 years or more experience. These older physicians confirmed that in some cases they did not inquire about the family history actively, because several members of the family attended their practice for many years, and the physician was very well aware about their family history.

*“After a long period of time you know the family history of all your patients. You may still ask for the family history to complete the record but after 30 years you know these things by heart. Finally it will not be done for each consultation, but it will be done in the course of time.” (participant 16)*

*“You must not forget that a family physician is usually the physician of the entire family. That means that people you have cared for as a child, now have their children. So you know the parents, the children and the grandchildren. In such families you should not ask for the family history.” (participant 14)*

Younger participants mainly payed attention to the family history in case of new patients, but they confirmed that the family history is certainly not inquired in every new patient or not systematically examined.

### 3.3. When Is the Family History Inquired?

The participants generally inquired about the family history during the first or one of the first consultations. They will inquire about the family history again if there is a clear indication for doing so. Almost none of the participants inquired the family history every year as a routine, even in the absence of suspected pathologies.

Participants generally stated that patients regularly consulted them with questions concerning their family (history). The most common questions asked by patients refer to malignancies, in particular breast and colon cancer. Some physicians reported on patients expressing concerns on the occurrence of psychological disorders, diabetes, coagulation disorders, and specific genetic syndromes or birth defects in their families. 

### 3.4. How Is the Family History Inquired about?

The physicians reported not to use a fixed questionnaire to inquire about the family history. Very few physicians used a more or less fixed list of questions, without the list being based on scientific recommendations. All physicians reported using open-ended questions such as “Are there certain diseases running in the family?”. Family histories are inquired when there is a clear reason to do so and open-ended questions are used focusing on the pathology the patient presents.

Some physicians reported to use the “Health Guide” of “Domus Medica.” None of the participants who used the “Health Guide” mentioned this spontaneously when asked for the use or not of a fixed questionnaire. 

The participants considered cardiovascular disease, cancer in general, and diabetes as the most important diseases in family history (Table 2).

Some participants reported to pay special attention to the family history of the parents in the case of an expected pregnancy. Familial occurrence of hip or eye defects and problems in pregnancy such as gestational diabetes also receive special attention.

### 3.5. Do You Only Ask Questions about First-Degree Relatives?

When the accuracy and breadth of family history was addressed, only few participants reported to go beyond the questioning of first-degree relatives. For some disorders they also involve second- and third-degree relatives. 

### 3.6. How Detailed Is the Family History Questioned?

When a patient has a family history for a particular condition, participants generally reported to ask additional questions on the occurrence of the condition in the patient's family.

They reported to inquire about the age of onset of the disease. Other elements that were reported were a brief outline of the disease process, age of death of the affected family member, and cause of death.

*“I will definitely ask about the age at which a condition is diagnosed. If that condition started very early, you need to focus on the patient at a younger age.” (participant 10)*

### 3.7. How Is the Family History Recorded?

Most participants reported to record the family history in the medical file, usually in the section “(family) history.” Only few physicians record data in the sections “medical problems” or “risk factors” using free text. Some participants declared to record the family history as free text without the use of special headings or to record the family history in the consultation reports using free text. None of the participants reported the use a family tree in the patient's medical file.

*“Sometimes I draw a family tree for myself, or during a consultation to visualise the family ties, but never a family tree is recorded in the file.” (participant 1)*

### 3.8. Medical Software

Most participants reported to use medical software instead of paper files. They declared not to be aware of the possibilities that their software packages could offer in terms of family history data.

*“Medical software companies could create a tool to integrate family trees in the medical record, or they could create a standard questionnaire." (participant 3) *

### 3.9. Negative Family History

Only few physicians recorded a negative family history when the patient confirmed the condition not to be present in his/her family. They all work in a duo or group practices. Most physicians reported not to record a negative family history despite that some of them work in a duo or group practice.

### 3.10. Importance of Family History

Most participants declared to believe that the family history is important to administer in family practice. Only few participants indicated that family history should receive more attention than it receives today.

Participants generally stated that family history is particularly important as it provides a broader medical and social picture of the patient and his relatives lives. They indicated the family history to be particularly useful for the prevention of various diseases.

*“For 2 reasons: there are more and more diseases where this is important, but the main reason is that we as a family physician are probably the only ones with a view on it." (participant 13)*

*“Sometimes, I must say, following a preventive consultation several times made surprising discoveries. So yes, actually it is more important than how it has hitherto been estimated.” (participant 12)*

The participants mainly had experience with the following familial disorders in their own practices: cardiovascular disease, colorectal pathology including colorectal cancer, and breast cancer. 

### 3.11. Risk Assessment

Most participants indicated that they certainly did not always feel capable to classify patients in high-, medium-, or low-risk groups. Very few participants often use official guidelines or instruments in order to determine the risk. Mainly the “Health Guide” from “Domus Medica” was indicated as an important source.

### 3.12. Management

Participants generally reported not to use official guidelines or instruments for the followup and treatment of patients at risk. They use their clinical intuition and experience to guide their patients or to decide upon referral to a medical specialist.

*“To my knowledge there are no, or I do not know where I can find, guidelines to classify patients into risk groups. I rather refer patients based on intuitive decisions.” (participant 9)*

*“I must admit that a lot of improvisation and intuition is used when a patient is referred, but that's the bulk of our work.” (participant 4)*

### 3.13. Reliability of the Family History

Participants stated that the information they receive from their patients on their family history is not always reliable. They considered that many patients do not have enough knowledge or information to conclude upon a reliable family history. Furthermore, some participants stated that patients exaggerate or provide irrelevant information. Only few participants indicated to believe that the information they get from their patients about their family is accurate.

*“Often they know that family members have problems with their intestines, but whether it is benign or malignant or whatever it was, they do not know. There are many who say that they do not know, but there are also those who say what they like or what they think it is.” (participant 12)*

### 3.14. (Personal) Improvements in Inquiring and Interpreting of Family History

Most physicians indicated that they will pay more attention to family history in the future and that they will inquire about family history on a more regular basis. Some physicians indicated that they would like to use a structured questionnaire to inquire about the family history.

Others reported that they could still make progress in terms of orderliness and structure whilst recording a family history.

*“And maybe I should also do more with it, it appears that I may ask the family history for certain patients, I just write it in their records and than nothing is done with it.” (participant 7)*

Many physicians believe that much progress is to be made in the field of information technology and the functionalities of electronic medical records. It was proposed that there should be an automatic notification in the medical records when a family member has an important condition.

Some physicians proposed a flowchart, diagram, or checklist with a limited number of specific questions for family history in the electronic record. It was also suggested that the family history should always be clearly visible when opening a file.

### 3.15. Obstacles in the Inquiry and Interpretation of Family History

Most participants indicated a lack of time as the main reason why they do not optimally record the family history. They noticed that the patient will probably not be keen to answering a long list of questions that is not related to their actual symptoms.

*“To inquire in each patient systematically the family history is asking too much! I think there should be a motive first. I think it makes sense to ask everyone's family history, but I do not see me doing this within my actual timeframe. I see 30 patients on a morning.” (participant 13)*

## 4. Discussion

### 4.1. Importance of Family History

This study shows that most family physicians consider family history as an important part of good clinical practice. This conclusion is in line with findings from other studies, both in Europe and the US [5, 16, 17, 24]. 

In the US, 63% of family physicians report to know the family history of at least three quarters of their patients, and 95% of these physicians inquire about the family history in a systematic way during the first visit of the patient [1, 26, 27]. In our study the family history is only updated if there is a clear reason to do so, which corresponds with data from other studies [16, 19].

Most family physicians acknowledge genetics and family history taking as an important aspect of primary care and the prevention or early diagnosis of multifactorial diseases. Unfortunately, clinical genetics are not identified as a specific learning outcome in the training of family physicians [13].

Geneticists suggest clinical genetics as a magic bullet that might prevent or cure many diseases. Family physicians have some doubts about this thesis [28, 29]. This seems also to be reflected in the results of our study. Family physicians seem to be concerned about anxiety produced by screening for disease which might not appear or which are not treatable [28]. This confirms the inconsistency between clinical genetic advances, family history use, and family physicians tasks in the prevention and early diagnosis of disease.

### 4.2. Inquiry of Family History

The way our participants made inquiries about family history differs greatly from the “gold standard” family tree that includes three generations. None of the physicians that were questioned in our study draw a family tree on a regular basis, and the family history is usually limited to the first-degree relatives. Although most physicians ask additional questions about family members, they often only ask about the age of onset of a particular condition. The completion of an extensive family tree requires substantial efforts, but also in the case of a less extensive family history, the lack of time is often perceived to be an important obstacle [1, 19, 24, 26] as was also reported by the physicians that participated in our study. The majority of participants also encountered problems with the reliability of the information they received from patients, which is also identified as a barrier for implementation in other studies [1, 16].

Another issue is the completeness of medical records. The absence of a risk factor or a negative family history is often not documented in patient records. This is not only a problem among physicians but also among nurses and other healthcare providers [30].

The surveyed physicians rarely or never use formal questionnaires or tools. This leads to the question to what extent such questionnaires are available in Belgium. In several studies a “family history tool” is used [1, 4, 8, 9, 12, 19]. In the review of Qureshi et al., 18 paper-based and 11 web-based tools were evaluated. Although many of these tools exist, they were not designed or adapted for use in primary care [8].

In Belgium, the best known preventive instrument is the “Health Guide” of “Domus Medica” that contains questions about the family history on heart disease, diabetes, breast and ovarian cancer, and colon cancer or polyps [24]. There is no consensus on which diseases should be included in a family history tool. However, the diseases included in the “Health Guide” are quite similar with the disorders that our participants considered as important. Although the Belgian physicians possess a tool for recording family history data, they do not consider the “Health Guide” to be a “family history tool.” This can in part be explained by the fact that the questionnaire—next to the family history—also contains various other items (including personal history and behaviour). Furthermore, the “Health Guide” was implemented among family physicians only a few months before our study; thus it is too early to evaluate its benefits in daily practice. Beside the “Health Guide” there are almost no other tools available in Belgium. However, the majority of physicians indicated that they prefer a more structured instrument to register the family history and they suggest incorporating such an instrument in their medical software. Very recently, several medical software companies developed a “family history tool” that was incorporated in their medical software. Unfortunately, many of them do not record second- and third-degree relatives. There is also a wish for more advanced features in the medical software, such as an autocomplete function that adds a familial risk factor for all family members.

The regular update of family history data is another issue. It is uncertain that patients themselves are always able to take the initiative to inform their physician about important disorders in their family. 

A study from the UK showed that in 40% of the patients who received a detailed family history, one or more diseases with a genetic component may be identified having an impact on the patient himself/herself or on his/her children [3]. This finding illustrates that many patients are not really aware of the familial risks, and they are probably not able to take initiative in informing their physician about changes in their family history. For these patients, a regular reviewing of the family history data by the family physician is a preferred option.

### 4.3. Interpretation of Family History

In addition to the collection and recording of family history data, our study also examined to what extent the physicians use the obtained information. The data are seen as the ideal way to identify individuals who, based on their family history, have an increased risk for certain diseases, but that implicates physicians to be able to appropriately estimate the risk for the patient.

A study by Wood et al. shows that physicians are quite uncertain about the interpretation of the family history of their patients. There is a demand for more guidelines and protocols for risk estimation [16]. Also a minority of the physicians in our study regularly used official guidelines for risk assessment. Several physicians admitted that their decision to refer patients for treatment or screening is rather intuitive, that is, more based on experience than on objective guidelines.

Although the majority of the participants admitted not being able to estimate the risk of their patients, they generally were quite confident to make appropriate referrals, based on a positive family history.

### 4.4. Limitations of the Study

Although the number of participants is comparable to that of similar studies, conclusions to this study are based on the opinions of a small group of physicians that may not be fully representative of the Belgian family physicians.

The participating physicians were all selected from the family medicine training supervisors of the Department of Family Medicine of the Vrije Universiteit Brussel, but most of them had no other special relationship with this university. In fact, most of them graduated at one of the other Belgian universities.

A limited number of participants responded at their own initiative to the first invitation by email. These participants might have had a special interest in the subject. However, the vast majority of participants were contacted by telephone, limiting a possible special interest in the research.

### 4.5. Future Research

Based on this study it seems important to further investigate the use of medical history in more detail. On the one hand further in-depth qualitative research is needed, and on the other hand quantitative research investigating patient records could provide interesting information about the use of family history in daily practice. From a review of patient records in nursing care we know that for such an audit different approaches exist such as formal structure, process comprehensiveness, knowledge base, and concordance with actual care [31]. 

Our study shows that the physicians were self-confident in determining the further management of patients with a positive family history, but they do not use scientific tools to determine the exact risk. In response to this, it should be investigated to what extent the decisions taken by physicians correspond to the existing guidelines. Further research about the use of tools and instruments for the assessment and interpretation of family history in primary health care is essential.

## 5. Conclusions

Family history can be used to identify patients with a higher risk for a certain disease and to propose for these patients an adapted follow-up procedure. The family physicians that participated in this study recognise the importance of family history in primary care, but they also encounter several barriers for an optimal inquiry about and interpretation of the family history. Many physicians experience a lack of time and a lack of reliable information from the patient. 

Physicians indicate the importance of structured tools and instruments to record family history data in their medical software.Only seldom tools or instruments for accurate risk assessment are used.

Most physicians prefer a more clinical intuitive, experience-based evaluation of patients at risk. However, some physicians ask for more guidelines for the classification of patients into risk groups.

## Figures and Tables

**Table 1:** Characteristics of the participants.

Physician				Practice		
No.	Gender	Experience as a family physician (in years)	Training in family history	Location	Number of physicians in practice + trainees	Particularities patient population
1	Female	4	No	Urban	7	Young population, many immigrants
2	Female	26	No	Rural	3	60% immigrants
3	Male	37	Yes	Urban	6 + 2	Young population, 20% immigrants
4	Male	26	No	Urban	1	
5	Female	4	Yes	Rural	2	
6	Male	10	No	Rural	4	Young population
7	Male	10	No	Urban	6	Many immigrants, many men
8	Female	23	No	Urban	3	Young population
9	Female	13	No	Rural	1	Young population
10	Male	19	No	Urban	2	Older population
11	Female	17	No	Rural	3	
12	Female	11	No	Rural	3	
13	Male	40	Yes	Urban	8	More than half Moroccan patients
14	Male	33	No	Urban	1	
15	Female	7	No	Rural	2	
16	Female	30	No	Rural	1	Young population, many women

**Table 2:** Diseases in which the family history is considered important by the participants.

Diseases	Number of physicians that consider family history as important for this condition (*N* = 16)
Cardiovascular diseases	13
Cancer (unspecified)	10
Diabetes	9
Breast cancer	5
Colon cancer	5
Hypertension	4
Thyroid disorders	3
Respiratory diseases	3
Genetic syndromes (unspecified)	2
Muscular diseases	2
Allergy	2
Skin disorders	1
Metabolic disorders	1
Manic-depression	1
Rheumatologic disorders	1
Ovarian cancer	1
Prostate cancer	1
Lung cancer	1
Cervical carcinoma	1
Cystic fibrosis	1
Hypercholesterolemia	1
Crohn's disease	1
Neurological disorders	1
Hemoglobinopathies	1
Blood diseases (leukemia, lymphoma)	1
Interstitial nephropathy	1
Social environment	1
